# Sustainable Starch-Based Biofilms Incorporated with Waste Hibiscus Flower Extract for Food-Packaging Applications

**DOI:** 10.1021/acsomega.6c07665

**Published:** 2026-09-11

**Authors:** Deepraj Sarkar, Ranjeet Kumar Mishra, Nagaraj Kamath H, Srinivas Kini

**Affiliations:** Manipal Institute of Technology, 76793 Manipal Academy of Higher Education, Manipal, India

## Abstract

The rapid increase in global demand for plastics has raised serious environmental and health concerns due to their nonbiodegradability and greenhouse gas (GHG) emissions. As a result, to mitigate these issues associated with traditional plastics, bioplastics emerge as a sustainable and promising solution. Therefore, the present study aims to develop starch-based bioplastic films incorporated with waste*Hibiscus rosa-sinensis* (*China rose*) flower extract (HFE) for sustainable food-packaging applications. The biofilm was fabricated using a combination of cornstarch, refined flour, citric acid, vinegar, and formaldehyde, with varying proportions of HFE, using the aqueous casting technique (Hb1, Hb2, Hb3, S). The results revealed that the biofilm HB1 exhibited moderate transparency, the highest TS (10.865 MPa), EB (14.57%), moderate YM (74.58 MPa), and a low MC (6.53%). The solubility tests revealed that the biofilms were stable in aqueous, acidic, basic, and alcoholic medium. FTIR analysis confirmed the presence of C–O, C=O, C–H, and a strong O–H peak, and WCA revealed moderate hydrophobicity. Finally, the degradation studies revealed that biofilm HB1 is more degradable. Further, the migration tests confirmed limited transfer of biofilm constituents into food simulants, indicating relatively low overall migration under the experimental conditions investigated. The incorporation of HFE into starch-based bioplastics serves as a bioactive additive, enhancing the physicochemical and functional properties of the biofilms by improving intermolecular interactions. This demonstrates their potential as sustainable alternatives to conventional petroleum-based plastics.

## Graphical abstract

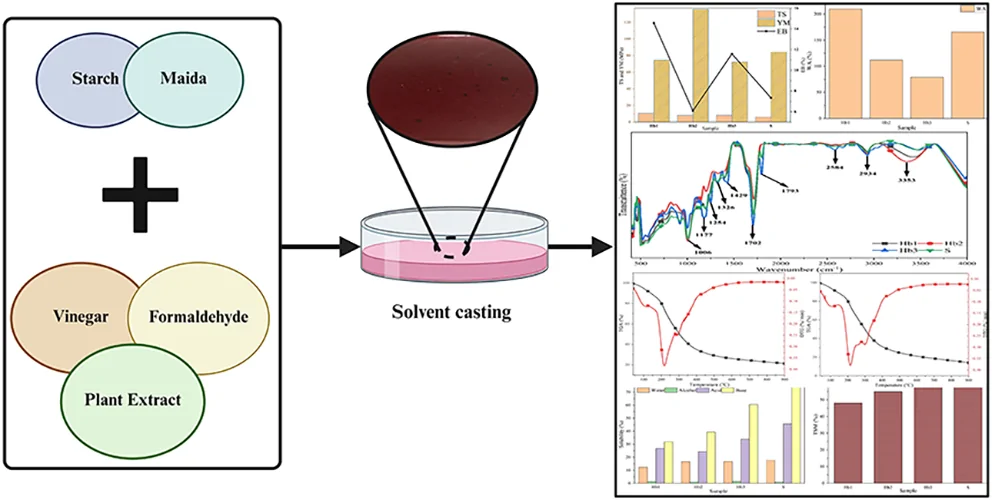

## 1 Introduction

Petroleum-based plastics are one of the most available synthetic materials, with production beginning just 70 years ago, yet reaching an estimated 9 billion metric tons by 2021.^1,2^ The excessive usage of single-use containers has significantly increased the production of plastics, making packaging the largest sector in the market.^3,4^ It is estimated that around 400 MT of plastics are produced worldwide annually, which generates 353 MT of waste, contributing 40% alone from the packaging sector.^5^ Plastics account for 90% of waste and are primarily derived from fossil hydrocarbons, which are depleting non-renewable resources and contribute to environmental pollution. Plastics are largely nonbiodegradable, which accumulate in landfills and produce various emissions.^6^ It is estimated that annually 4–12 MT of plastic waste enters the oceans, where it fragments into microplastics, affecting the marine ecosystems.^6,7^ The presence of plastic debris in ocean basins and the environment is so widespread that it can serve as a geological indicator of the *anthropocene*. Although recycling is proposed to mitigate plastic waste, due to the high costs, technical limitations for different polymer types, and poor quality of the obtained recycled materials, it limits it to less than 9% of global plastic waste generation.^8^ Therefore, growing concerns about the accumulation and disposal of traditional plastics, along with the implementation of strict global regulations, are driving the plastic industry toward sustainable alternatives. Bioplastics derived from renewable sources, such as starch and lignocellulosic biomass, have emerged as a promising solution to petroleum-based plastics. It has the potential to reduce the environmental impact while meeting the functional material needs of various industries.

Starch is considered the most sustainable material to produce bioplastics because of its renewability, biodegradability, and compatibility with processing techniques such as extrusion, injection, and compression molding. Despite these advantages, starch-based bioplastics exhibit inherent demerits like low water resistance and mechanical and thermal properties.^9,10^ The inclusion of lignocellulosic fillers has proven to be an effective approach to enhancing these properties. Lignocellulosic materials include water hyacinth,^11^ red algae,^12^ bagasse fiber,^13^ pineapple leaf fiber,^14^ rice husk, and floral waste.^15^ They are remarkably favorable due to their nontoxicity, high degradability, low density, and chemical similarity to starch. Furthermore, the interaction between the starch matrix and lignocellulosic-derived fillers promotes strong interfacial bonding, enhancing the material’s overall performance. Additionally, using agricultural residues minimizes waste generation and offers economic and environmental advantages by reducing the need for landfill disposal of biomass.^9^

In India, most of the flower waste is generated from religious offerings, celebrations, and banquet functions, contributing to around 300 tons of flower waste.^16^ Flowers such as marigolds, roses, and hibiscus (China rose) are often discarded improperly from temples, roadside stalls, and event venues. It is estimated that 8 MT of flowers are offered annually at various religious sites and are later discarded into rivers and landfills. These discarded flowers or waste flowers pose a serious threat to the ecosystem.^16^ Moreover, it releases high levels of organic matter due to the presence of flower waste that can accelerate algal growth in water bodies. The increased growth of algal bodies in wastewater reduces the oxygen content in the water, which affects the marine ecosystem.^16^ Furthermore, roadside discarded waste attracts flies and mosquitoes, which can spread many diseases. Also, it degrades the quality of the air because of the emission of GHGs like CO_2_ and CH_4_ from the decaying flower waste.^17^ Furthermore, floral waste can be effectively utilized as a renewable resource by isolating and extracting its components, which can then be incorporated into bioplastics to produce a sustainable composite material.

Several studies have reported on the fabrication of biofilms using plant-based natural extracts. Sedyadi and Yuliati (2020) produced bioplastics using *sirih leaf* extract and canna starch and found the thickness was 0.083 mm, TS of 3.11 MPa, EB of 27.62%, and YM of 0.11 MPa.^18^ Oluwasina and Awonyemi (2021) produced bioplastics based on cassava starch and citrus lemon ethanol extract (CLPEE) (0–20%). The results showed that with a rise in the amount of extract, the MC reduced from 14 to 12%, S_Water_ reduced from 2.84 to 1.23%, and TS increased from 9 to 25 MPa.^19^ Nasution et al. (2021) developed bioplastics using sago starch and betel leaf extract (3–9%). The results indicate that the addition of extract reduces the WA from 80 to 36% and inhibits the activity of bacteria (*Bacillus cereus*).^20^ Santos et al. (2025) fabricated bioplastics based on cassava starch and *vismia guianensis* alcoholic extract (EAVG) (0.2–1%). Krishnamurthy and Amritkumar (2019) produced bioplastics from PLA and starch, along with lemon extract, glycerol, and palm oil. The results showed that WA was between 33–285%, *S*_Water_ (12–70%), TS (0.7–5.2 MPa), EB (3–73%).^21^ Elsaeed et al. (2023) developed bioplastics based on poly­(vinyl alcohol)/gellan gum, incorporating Psidium guajava (guava) leaves and chickpea extracts. The results revealed that the addition of guava leaves and chickpea extract increased the TS from 0.18 MPa to 0.21 and 0.26 MPa, whereas WCA decreased from 87° to 56 and 72°. The WVTR test did not show any significant changes (5.7 g/m^2^/d), but the OP increased from 18 to 43 and 67 g/m^2^/s.^22^ The literature review found that no study has been conducted on biofilms using a *Hibiscus rosa-sinensis*flower-based extract, to the best of author’s knowledge. However, previous studies have reported that *H. rosa-sinensis*­(China rose) contains a wide range of bioactive compounds (phenolic compounds, flavonoids, tannins, alkaloids, terpenoids, and other phytochemicals) that exhibit antioxidant and antimicrobial activities.^23,24^ Accordingly, the present work employed Hibiscus extract, although the phytochemical composition and bioactivity of the extract were not experimentally characterized. Therefore, incorporating flower extract into bioplastics is being explored as a viable strategy, as these bioactive compounds improve material properties while also contributing to the sustainable management of floral waste.^25^

In light of the aforementioned research gap, the present study aims to develop a degradable biofilm composed of starch and waste Hibiscus flower extract (HFE) for food-packaging applications. Corn starch, refined flour, HFE, formaldehyde, citric acid, and vinegar were utilized in varying proportions as raw materials. Corn starch was employed as the primary film-forming polymer, with refined wheat flour serving as a starch-rich co-matrix to improve processability and structural integrity. *Rosa sinensis* extract functioned as an antimicrobial and crosslinking agent. Additionally, formaldehyde, citric acid, and acetic acid contributed as plasticizers and crosslinking agents. However, formaldehyde was used only as a laboratory-scale chemical crosslinking agent to evaluate the effect of increased crosslink density on the physicochemical properties of starch-based biofilms. Its use was intended for fundamental material development and should not be interpreted as a validation for direct food-contact packaging applications. The resulting biofilms were characterized using various analyses, including TGA, FTIR, WCA, tensile testing, Young’s modulus, water absorption, moisture content, density, solubility, and degradation studies. The developed starch-based biofilm contributes to several United Nations Sustainable Development Goals (SDGs), including SDG 3 (Good Health and Well-being) by providing a safer and biodegradable alternative to petroleum-based packaging materials, SDG 6 (Clean Water and Sanitation) by reducing plastic and floral waste pollution in water bodies, SDG 13 (Climate Action) through the use of renewable resources and biodegradable materials that help lower greenhouse gas emissions and dependence on fossil-derived plastics, SDG 14 (Life Below Water) by minimizing plastic leakage and microplastic contamination in aquatic ecosystems, and SDG 15 (Life on Land) by promoting the valorization of waste flowers into value-added bio-based packaging materials, thereby reducing landfill disposal and supporting sustainable resource management.

## 2 Materials and Methodology

### 2.1 Preparation of Sample

Waste hibiscus flowers (*H. rosa-sinensis*) were sourced from a nearby temple in Udupi, Karnataka, India. The samples were thoroughly rinsed with distilled water to remove surface impurities, then sun-dried for 24–42 h. Following this, the partially dried samples were heated in a hot-air oven for 2–3 h at 105 °C to uniformly remove moisture. The fully dried material was subsequently pulverized using a mixer grinder to obtain particles in the size range of 800 μm to 1 mm, and the powder was stored in an air-tight bag to prevent moisture absorption.

### 2.2 Preparation of Flower Extract

HFE was prepared by homogeneously dispersing hibiscus powder in distilled water at 10% (w/v) using a 500 mL cylindrical beaker. The mixture was continuously stirred for 5 min at 100 °C. Afterwards, the resultant solution was permitted to cool to room temperature and subsequently stored at 4 °C in a refrigerator for further use in bioplastic production. However, the extraction procedure was intentionally designed as a simple aqueous extraction to directly utilize waste hibiscus flowers without solvent-intensive purification and drying. Accordingly, the extract was incorporated in its semi-liquid (paste) form, and the concentration, freeze-drying, and spray-drying steps were not performed. Further, the total phenolic content, antioxidant capacity, antimicrobial activity, and detailed phytochemical composition of the extract were not experimentally determined in the present study but were inferred from published literature on *H. rosa-sinensis*.^23,24^ Since the extract was incorporated directly in its aqueous form without concentration and drying, the extraction yield and dry solid content were not determined. Consequently, the extract loading reported in Table 1 corresponds to the mass of the aqueous extract rather than the dry extract mass.

**Table 1:** Formulation Ratios of Biofilms From Starch, HFE, Plasticizers and Other Raw Materials^a^,^b^

sample name	cornstarch (g)	refined flour (g)	citric acid anhydrous 99.5 % (g)	acetic acid (mL)	HFE (g)	formaldehyde (37%) (mL)
S	5	5	12.5	55		15
Hb1	5	5	7.5	45	7.5	5
Hb2	5	5	10	50	10	10
Hb3	5	5	12.5	55	12.5	15

a Hb1, Hb2, and Hb3 represent sets 1, 2 and 3.

b The HFE values listed represent the masses of the freshly prepared aqueous extract added to the formulation.

### 2.3 Formulation of Biofilms

The biofilm was prepared employing cornstarch, refined flour, *H. rosa-sinensis* extract, citric acid, acetic acid, and formaldehyde in varying formulations. Corn starch served as the primary film-forming biopolymer, whereas refined wheat flour functioned as a starch-rich co-matrix. Although equal masses of corn starch and refined flour were used, refined flour consists predominantly of starch (70–75 wt %) and includes gluten proteins, lipids, and minor constituents. However, the continuous polymeric network remains starch-dominated, while the gluten fraction contributes to film cohesion, gelatinization behavior, and mechanical integrity through additional intermolecular interactions. However, based on previous phytochemical reports for *H. rosa-sinensis*, the aqueous extract is expected to contain phenolic compounds, flavonoids, and tannins. Therefore, it contributes to hydrogen bonding with the starch matrix and improves film performance. However, these constituents were not quantitatively analyzed in the present study. Furthermore, these components promoted intermolecular hydrogen bonding within the starch matrix, thereby enhancing the mechanical strength and overall functional performance of the biofilm. Citric acid was incorporated as a bio-based crosslinking agent and a secondary plasticizer. Its tricarboxylic structure enables ester bond formation with the −OH groups of starch, strengthening the polymer network, improving tensile strength, and lowering moisture sensitivity.^26^ Vinegar (acetic acid), on the other hand, served as a mild, environmentally friendly plasticizer and pH regulator, aiding starch gelatinization and improving flexibility. The presence of acetic acid and water in vinegar facilitates plasticization by allowing small molecules to diffuse between polymer chains, weakening intermolecular forces and increasing chain mobility without forming permanent linkages. Additionally, citric acid contributes to plasticization through hydrogen-bonding interactions between its hydroxyl and carboxyl groups and the hydroxyl groups of starch. This interaction weakens polymer-chain associations, reduces rigidity, and enhances molecular mobility, thereby improving elongation at break and flexibility. A small quantity of formaldehyde (37%) was incorporated solely as a laboratory-scale crosslinking agent to investigate the effect of increased crosslink density on the starch polymer network. Formaldehyde promotes the formation of methylene bridges between hydroxyl-rich polymer chains, thereby improving structural integrity, tensile strength, moisture resistance, and thermal stability. The objective of its inclusion was to establish a proof of concept regarding the influence of chemical crosslinking on starch-based biofilms, rather than to develop a commercial food-contact material. However, owing to the well-recognized toxicity of formaldehyde and current food-contact regulations, the developed films are intended only for secondary packaging applications. The composition ratios of the biofilms for different formulations are presented in Table 1. The prepared mixture was then stirred using a magnetic stirrer at 30 °C at 1000 rpm until a homogeneous solution was obtained. Subsequently, the solution was cast onto a glass tray and dried in an oven at 70 °C for 15 h. After the biofilms dried, they were carefully peeled off and placed in a desiccator for further investigation. Furthermore, the present formulations were developed as integrated compositions in which the concentrations of HFE, citric acid, acetic acid, and formaldehyde were varied simultaneously to achieve balanced film-forming and crosslinking characteristics. Consequently, the experimental design was intended to compare the overall performance of different formulations rather than to isolate the individual contribution of hibiscus extract. Therefore, the observed physicochemical and mechanical properties should be interpreted as the combined effect of all formulation components.

### 2.4 Biofilm Characterization

#### 2.4.1 Physical Characterization

The MC of the prepared biofilm was evaluated in accordance with ASTM D6980, with minor modifications. A 2 cm^2^ sample was weighed on a digital balance and then dried in a hot-air oven (Rotek (RHO-18HDFSS)) at 105 °C for 3 h. After drying, the sample was weighed again, and the MC was determined from the weight difference. The thickness of the biofilm was evaluated using 2 cm^2^ sample, and thickness readings were recorded at 15 different locations using a vernier calliper. Furthermore, the density of the prepared biofilm was calculated by dividing its mass by its volume. For transparency analysis, a 5 cm^2^ biofilm sample was placed on a desktop screen set to 300 nits. The word “transparency,” displayed in Times New Roman, size 48, was used as the reference for visual evaluation.

#### 2.4.2 Mechanical Characterization

The mechanical performance of the biofilms was assessed using specimens measuring 60 × 20 mm for TS, EB, and YM. The analysis was performed using a UTM (Shimadzu Ez-Sx) equipped with a 500 N load and operated at a crosshead speed of 8 mm/min, in accordance with ASTM D882-12, with minor modifications. The TS was determined by dividing the max load at failure by the sample cross-sectional area. Elongation at break was calculated based on the extension in the initial gauge length at the moment of rupture, while YM was calculated using the slope of the stress/strain curve, representing the ratio of stress to strain.

#### 2.4.3 Solubility Test

The prepared biofilms were assessed for their solubility indifferent medium, which include alcohol (99% ethanol), an acidic solution (10% H_2_SO_4_, v/v), distilled water, and an alkaline solution (10% NaOH, w/v). A 2 cm^2^ of the biofilm was first dried in an oven for 24 h at 105 °C to determine its initial weight. The dried sample was then immersed in 50 mL of each medium and kept at room temperature for 24 h. Following immersion, the biofilms were re-dried in the oven to obtain the final dry weight. Solubility was determined by the difference between the initial and final weights. All measurements were performed in duplicate to ensure reliability.

#### 2.4.4 Water Absorption Test and Chemical Resistance Test

The water absorption (WA) test was performed in accordance with ASTM D570-98, with minor modifications. A 2 cm^2^ biofilm sample was initially dried in an oven at 105 °C for 24 h to evaluate its initial weight. The dried sample was immersed in 50 mL of distilled water and incubated at room temperature for 24 h. The WA was measured based on the change in weight before and after immersion. All measurements were performed in duplicate to ensure reliability. The chemical resistance (CR) test was evaluated to determine the influence of chemical exposure on the sample’s physical characteristics under acidic (10% HCOOH, v/v) and alkaline (10% NaOH, w/v) conditions. A 2 cm^2^ biofilm sample was immersed separately in both solutions for 24 h at room temperature. Solvent resistance was determined by monitoring any visible physical changes, including discolouration.

#### 2.4.5 Total Soluble Matter (TSM)

The TSM was measured by using a biofilm sample (2 cm^2^), which was initially weighed using a digital balance. It was subsequently immersed in 50 mL of distilled water and kept for 24 h at room temperature. Afterward, the remaining undissolved film was collected and oven-dried to obtain its final dry weight. The TSM was calculated on the basis of the difference in weights. All of the experiments were performed in duplicate for reproducibility.

#### 2.4.6 Migration Test

The biofilm was subjected to a migration test in accordance with the EU Directive 82/711/EEC. To assess the overall transfer of substances from biofilms into food, 5 food simulants were used, as shown in Table 2. A biofilm sample (3 cm^2^) was placed in 50 mL of each simulant and stored at 4 °C for 10 days. After the incubation period, the maximum absorbance (λ_max_) was recorded utilizing a UV–visible spectrophotometer to measure the level of migrated compounds.

**Table 2:** Determination of Migration in Various Food Types Using Various Simulant Solutions

simulant solution number	food mimicking simulant (V/V)	various types of food
1	distilled water	aqueous (pH > 4.5)
2	3% vinegar	acidic (pH ≤ 4.5)
3	10% ethyl alcohol	alcoholic
4	95% ethyl alcohol	fatty
5	50% ethyl alcohol	dairy products

#### 2.4.7 Scanning Electron Microscopy

The surface morphology of the starch and HFE based biofilm was analyzed using scanning electron microscopy (ESM, Zeiss-EVO MA18 with Oxford EDS). A small piece of the film was mounted on a specimen holder and sputter-coated with a thin layer of gold by using a precision vacuum sputter coater to improve electrical conductivity. The SEM was operated at an accelerating voltage of 15 kV.

#### 2.4.8 FTIR Analysis

A FTIR-ATR analyzer (Bruker α II) was employed to analyze the functional groups present in the biofilm over the wavenumber range of 4000–400 cm^–1^ at 64 scans with a resolution of 4 cm^–1^. Prior to analysis, the prepared biofilm was oven-dried for 3 h at 105 °C to remove any free moisture.

#### 2.4.9 Thermal Analysis (TGA) and Water Contact Angle (WCA)

The thermal breakdown behavior of the prepared samples was analyzed using TGA (PerkinElmer STA 6000 with Auto Sampler (Software: Pyris)) under an N_2_ atmosphere. Approximately 5 mg of the biofilm was placed in a ceramic pan and heated at a rate of 10 °C/min from 30 to 900 °C. The water contact angle (WCA) was measured employing a contact angle goniometer (Holmarc Opto-Mechatronics Ltd.) on biofilm samples (10 × 50 mm) at 25 °C. A 10 μL droplet of ultrapure distilled water was gently deposited onto the biofilm surface, and images were documented immediately. The experiments were performed thrice for each formulated biofilm.

#### 2.4.10 Assessment of Biofilm Degradation

The degradation analysis was carried out in various environments, including sand, garden soil, and water sources (distilled, ocean, and tap water). A biofilm sample (2 cm^2^) was weighed using a digital balance, then buried 2–4 cm deep in soil and sand within a gardening pot while maintaining moisture. The films were retrieved at 5-day intervals, cleaned, and reweighed. For water-based degradation, biofilm samples (2 cm^2^) were immersed in 50 mL of each water type, and mass changes were measured every 5 days. The entire procedure lasted 30 days, and decomposition was assessed by weight loss in each environment. For photodegradation analysis, a biofilm sample (3 cm^2^) was weighed and subjected to sunlight for 7 h daily at 28–33 °C under rain-free conditions. This test was performed over 14 days, and degradation was analyzed by measuring weight changes. The experiments were repeated twice for the best accuracy.

## 3 Results and Discussions

### 3.1 Physical Characterization

Moisture content, thickness, density, and transparency of the developed samples were analyzed against standard requirements to determine their suitability for food-packaging applications. Figure 1 illustrates the biofilms developed using starch and HFE, along with a control film prepared without any flower extract. Figure 2 illustrates the transparency assessment of the various biofilms, highlighting their potential use in sectors where product visibility and light transmission are important. The MC values for biofilms Hb1, Hb2, Hb3, and S were recorded as 6.537 ± 0.938, 9.116 ± 1.059, 9.315 ± 0.866, and 8.5925 ± 1.267%, respectively (Table 3). The lower MC of Hb1 can be attributed to an optimal crosslink density achieved by formaldehyde, together with strong hydrogen-bonding interactions between starch and hibiscus-derived phenolic compounds, resulting in a compact polymer network with fewer accessible hydroxyl groups for moisture adsorption. Although formaldehyde reduces moisture uptake through chemical crosslinking, the higher MC observed in Hb2 and Hb3 indicates that excessive extract and citric acid introduce additional hydrophilic groups (as confirmed by FTIR results) that partially offset this effect. The obtained results for MC (6–9.5%) are in close agreement with previous literature (5–17%).^3,19,27^ Similar MC have been reported where they used waste *Colocasia esculenta* (taro) starch, citrus peel extract and cornstarch with fructose, glycerol, and their combination as plasticizers. The MC generally ranges from 5 to 17%, depending on plasticizer content and filler composition. However, compared to these studies, the Hb1 formulation exhibited comparatively lower MC, indicating that the optimized concentration of hibiscus extract promoted stronger intermolecular hydrogen bonding within the starch matrix. Further, the differences among studies may also arise from variations in starch type, extract chemistry, drying conditions, and crosslinking density, all of which may influence the availability of free hydroxyl groups for water adsorption. The biofilm thickness was maintained at 0.1 cm. However, the densities were 0.733, 0.740, 1.020, and 0.808 g/cm^3^ for Hb1, Hb2, Hb3, and S, respectively. The density analysis showed that Hb3 had the highest density due to close molecular packing of higher solids and crosslinking materials, leading to higher −OH concentration and higher MC. The transparency assessment revealed that transparency decreased as hibiscus extract concentration increased. Hb1 exhibited the highest transparency among the films that contain extract, followed by Hb2, whereas Hb3 showed the lowest transparency. This behavior is attributed to the higher concentration of anthocyanins, flavonoids, and other phenolic pigments in Hb3, which increases visible-light absorption and internal light scattering within the polymer matrix. In addition, the higher solids content and denser microstructure of Hb3 further reduce light transmission, resulting in an opaquer appearance. As expected, the control film (S), which contained no colored phytochemicals, exhibited the highest transparency. The films demonstrated promising potential as food-packaging materials, with lower MC recommended for food safety and high transparency supporting consumer appeal.

**Figure 1:**
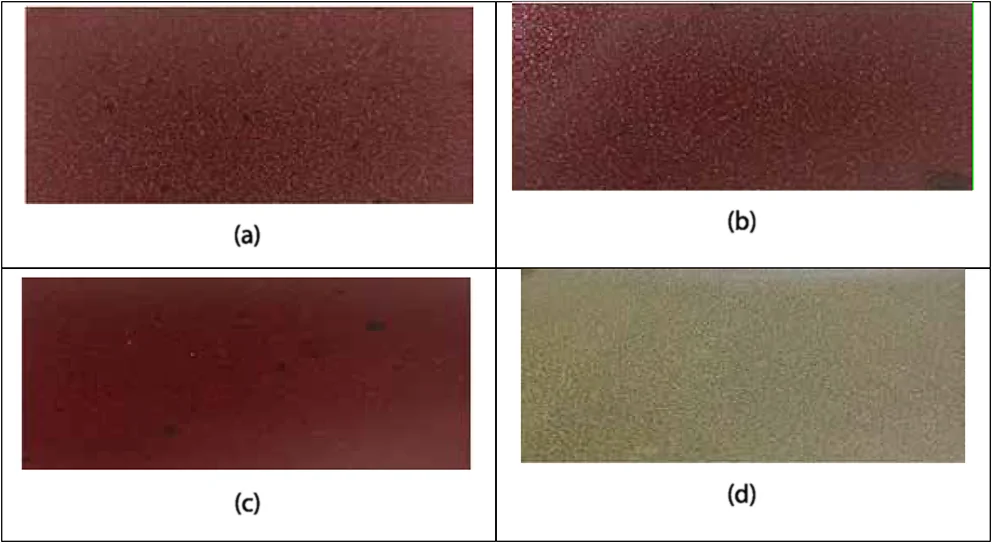
Biofilm production using starch and HFE. (a) Hb1, (b) Hb2, (c) Hb3, (d) S.

**Figure 2:**
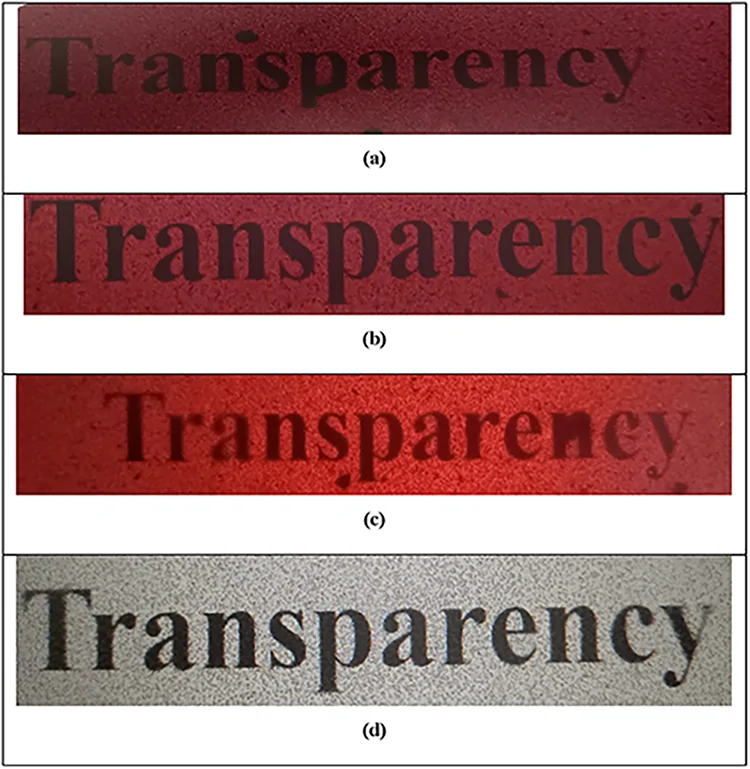
Transparency of the prepared biofilms (a) Hb1, (b) Hb2, (c) Hb3, (d) S.

**Table 3:** Moisture Content, Thickness, Density, and Color Characteristics of the Developed Biofilms

sample	color	MC (%)	thickness (cm)	density (g/cm^3^)
Hb1	red	6.537 ± 0.938	0.1	0.733
Hb2	red	9.116 ± 1.059	0.1	0.740
Hb3	red	9.315 ± 0.866	0.1	1.020
S	off-white	8.592 ± 1.267	0.1	0.808

### 3.2 Mechanical Characterization

Mechanical testing is crucial for ensuring that food-packaging materials withstand demanding processing and transportation conditions. Therefore, the tensile strength (TS), elongation at break (EB), and Young’s modulus (YM) of the developed biofilm were analyzed to assess its potential applicability in the food-packaging sector (Figure 3a). The TS of Hb1, Hb2, Hb3, and S are 10.865, 8.246, 8.377, and 6.187 MPa, respectively whereas YM was 74.581, 135.692, 72.297, and 84.235 MPa for Hb1, Hb2, Hb3, and S. The EB was found to be 14.568, 6.077, 11.587, and 7.341% for the prepared biofilm Hb1, Hb2, Hb3, and S. The mechanical characterization demonstrated that Hb1 had the highest TS and EB which suggests that the addition of extract with starch formed strong hydrogen bond and ester linkage (supported by FTIR) making the biofilm stronger without disturbing the starch chain structure. Although HFE contains hydroxyl-rich phytochemicals capable of forming hydrogen bonds with starch, increasing the extract concentration does not necessarily produce a proportional increase in mechanical strength. At the optimum concentration (Hb1), hydrogen bonding enhances interfacial cohesion and stress transfer. Beyond this level (Hb2 and Hb3), excess extract introduces low-molecular-weight hydrophilic compounds that increase polymer-chain mobility and free volume, while promoting partial aggregation within the matrix (supported by SEM). Consequently, the plasticization and structural heterogeneity offset the reinforcing effect of hydrogen bonding, resulting in lower TS. Similarly, the nonlinear variation in YM reflects the competing influences of crosslink density, extract dispersion, and polymer-chain mobility rather than experimental inconsistency. Apart from that, crosslinkers and plasticizers also play a key role in the better mechanical properties of the prepared biofilm. In Hb2, the highest YM and the lowest EB indicate a stiff, brittle structure that restricts polymer mobility due to excess crosslinking agents such as citric acid, extract, and formaldehyde. Hb3 showed moderate TS and EB with low YM, as the addition of higher HFE content helped maintain flexibility. However, the increased formaldehyde concentration resulted in a highly cross-linked, hardened polymer network. Furthermore, excessive crosslinking restricted polymer-chain mobility, generated internal stresses, and increased brittleness. Therefore, the excessive addition reduces the matrix’s ability to effectively transfer and dissipate applied stress. Consequently, premature crack initiation under tensile loading resulted in a lower TS than in Hb1. Further, the biofilm S lacks reinforcement, showing the lowest TS and EB, confirming the role of extract-derived functional groups. The findings for tensile strength (6–12 MPa), EB (7–19%), and YM (48–164 MPa) are consistent with values reported in the literature, where TS ranged from 1–4 MPa, YM from 2–72 MPa, and EB was 88%.^28–30^ Similar mechanical properties have been reported using Avocado, Jackfruit, and Durian starch with seaweed; yam starch with bentonite; and cornstarch with titanium dioxide NPs. The mechanical characterization reported generally ranges from 1–4 MPa for TS, 2–72 MPa for YM, and 88% for EB. However, compared with this literature, Hb1 exhibited higher TS while maintaining moderate flexibility. The improved mechanical performance can be attributed to efficient stress transfer through hydrogen bonding between starch chains and phenolic constituents present in the HFE. In contrast, studies employing excessive extract loading and weaker polymer/filler compatibility generally reported lower TS because of particle aggregation and poor interfacial adhesion. These observations indicate that optimization of extract concentration is critical for balancing strength and flexibility.

**Figure 3:**
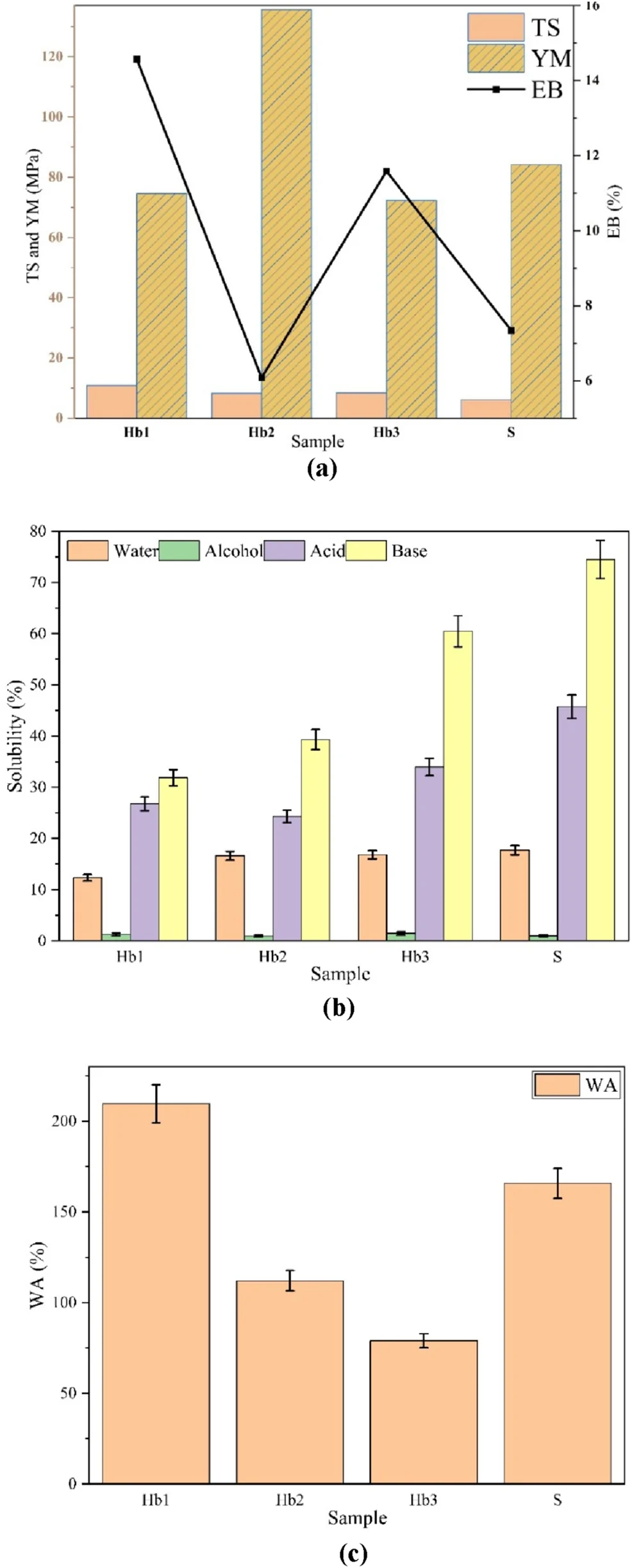
(a) Mechanical characterization (tensile, elongation, and Young’s modulus of the prepared biofilms); (b) Solubility behavior of the biofilms in water, acidic, alcohol, and basic media; (c) WA capacity of the prepared biofilms.

### 3.3 Solubility Test

The solubility test is a critical parameter for assessing the suitability of food-packaging materials as it indicates their resistance to moisture and, by extension, their ability to prevent food spoilage. The higher solubility suggests that the material may dissolve upon exposure to moisture, compromising food integrity, whereas lower solubility reflects improved resistance and enhanced protection against spoilage.^31,32^ Figure 3b illustrates the solubility behavior of the prepared biofilms in water, alcohol, acidic, and alkaline medium. Further, as the concentrations of HFE, citric acid, acetic acid, and formaldehyde were varied simultaneously across the formulations, the differences in solubility are attributed to the combined influence of extract incorporation, crosslink density, and plasticization rather than to the extract alone. The solubility of the biofilms was evaluated in water (*S*_water_), alcohol (*S*_alcohol_), acidic (*S*_acidic_), and basic (*S*_basic_) solutions. For Hb1, the solubility values were 12.352, 1.291, 26.755, and 31.878%, respectively. For Hb2, the corresponding values were 16.636, 0.970, 24.305, and 39.309%. Hb3 exhibited solubility values of 16.805, 1.482, 33.971, and 60.487%, while sample *S* showed 17.717, 0.980, 45.731, and 74.491% in water, alcohol, acidic, and alkaline medium, respectively. The solubility test revealed that Hb1 had the lowest *S*_water_ due to a cohesive, well-bonded polymer network that restricts moisture, whereas Hb2 showed the lowest *S*_alcohol_ and *S*_acidic_, suggesting superior solvent resistance due to a dense, crosslinked structure. Hb3 showed the highest solubility in (*S*_acidic_) and (*S*_basic_), revealing weaker chemical stability of the polymer structure in extreme pH, whereas biofilm S showed the highest solubility in (*S*_acidic_) and (*S*_basic_), which is consistent with the hydrolytic susceptibility of starch. The results obtained for water and alcohol solubility are consistent with those reported in the literature, which indicate water solubility in the range of 30–33% and alcohol solubility between 17–33%.^33^

### 3.4 Water Absorption (WA) Test and Chemical Resistance (CR) Test

The WA test is essential for evaluating the resistance and moisture diffusivity of biofilms under humid environments, thereby indicating their stability for food-packaging applications. Figure 3c shows the WA behavior of the developed biofilms, with Hb3 exhibiting the lowest absorption, followed by Hb2, S, and Hb1. The WA values were 209.642, 112.066, 79.001, and 165.797% for Hb1, Hb2, Hb3, and S, respectively. The WA tests exhibited different behaviors where Hb1 demonstrated the highest WA, likely due to its surface OH group, which facilitates rapid binding, although it has low MC. In contrast, Hb3 adsorbed the least amount of water due to its high density, which limits diffusion. Although the WAs are relatively high due to the inherently hydrophilic nature of the starch matrix, the values obtained remain within the range reported for starch-based biodegradable films. The progressive reduction in WA from Hb1 to Hb3 demonstrates that increased crosslink density and a denser polymer network effectively restricted water diffusion. Therefore, the developed films are more appropriate for secondary packaging or for dry/low-moisture food applications than for the direct packaging of high moisture foods. Further improvements in moisture resistance can be achieved in future studies by incorporating food-grade hydrophobic additives and alternative crosslinking strategies. The WA findings (79–209%) were comparable to the existing literature (60–430%).^34–36^ Similar WA properties have been reported for systems using potato starch with sorbitol and glycerol; banana peel starch; and a composite of banana peel starch, cornstarch, and rice starch, with potato peel powder and wood dust powder as fillers. The WA generally ranges from 60–430% in the reported literature. However, the relatively low WA of Hb3 indicates that increased polymer packing and higher crosslink density effectively restricted water diffusion into the matrix.

The CR of food-packaging materials is an important parameter for assessing the structural integrity and functional stability of biofilms when they are exposed to acidic and alkaline environments. This property ensures that the packaging retains its barrier performance, thereby protecting food and prolonging shelf life. Figure 4a–d illustrates the CR of the developed biofilms in an acidic medium, while Figure 4e–h represents their behavior in a basic solution. The results indicate a clear difference in the biofilm performance under acidic and alkaline conditions. In the acidic medium, the biofilms showed no noticeable change in solution transparency, likely because of the stability of the anthocyanins and phenolic compounds present in the HFE. These compounds remain stable in their flavylium cation form under acidic conditions.^37^ Additionally, citric acid enhances the starch matrix through esterification, thereby reducing pigment leaching and maintaining the structural integrity of the films. In contrast, significant changes in the solution transparency were observed in the basic medium, suggesting poor compatibility under basic conditions. This behavior can be explained by the pH-responsive nature of the pigments, which undergo structural transformations from the flavylium cation to quinoidal base and chalcone forms, resulting in significant color fading.^38^ Furthermore, partial hydrolysis of the ester linkages in starch and citric acid, along with weakening of methanal-induced cross-links, exposes the pigments and promotes their degradation. The results demonstrate that the biofilms possess strong chemical resistance in acidic environments, whereas their structural and color stability is considerably reduced under alkaline conditions. However, the trends observed in water absorption and chemical resistance reflect the overall formulation chemistry, including variations in crosslinking and plasticization, and should not be interpreted as the isolated effect of HFE.

**Figure 4:**
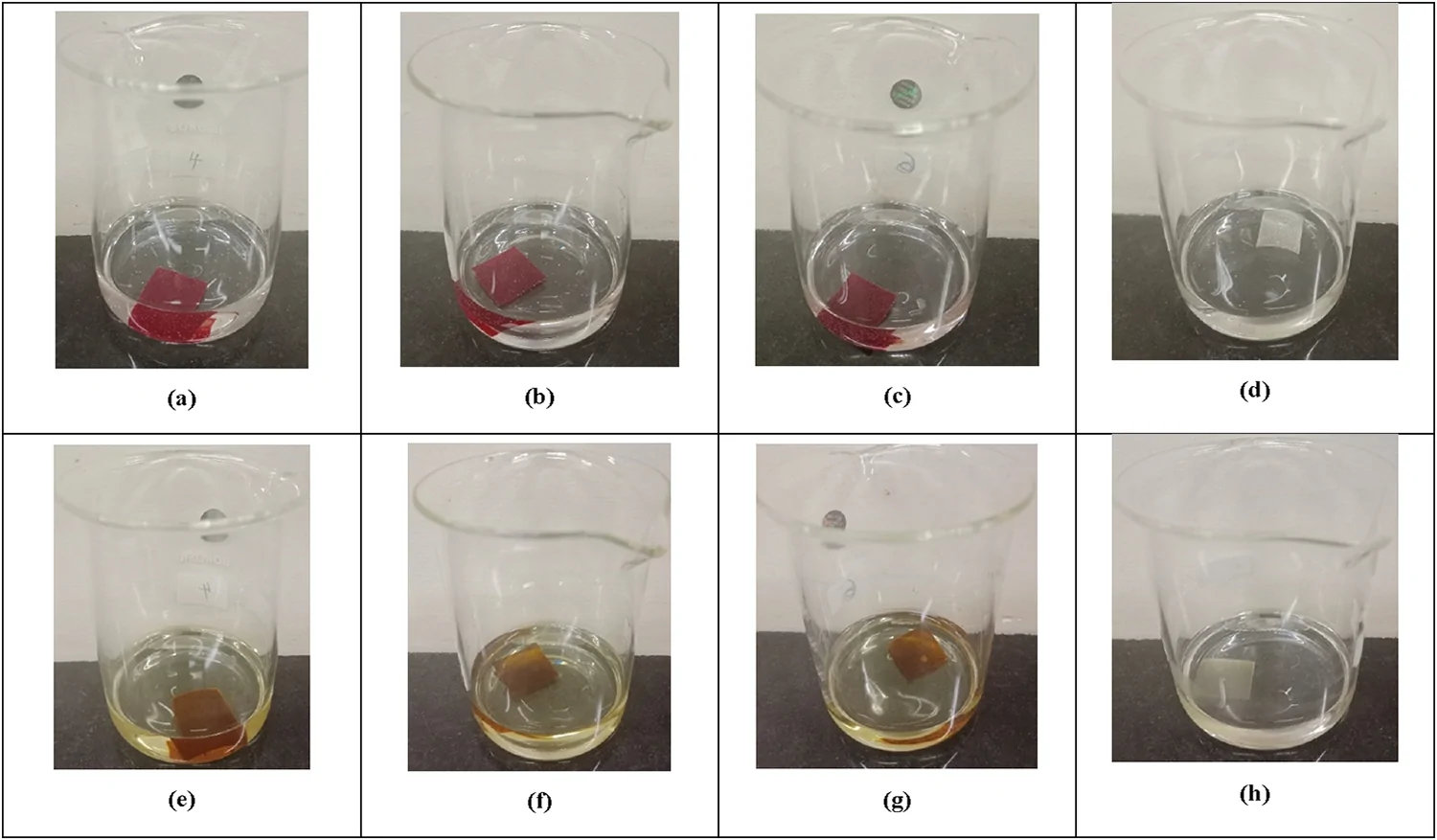
CR of the prepared biofilms evaluated in acidic medium: (a) Hb1, (b) Hb2, (c) Hb3, (d) S; and in basic medium: (e) Hb1, (f) Hb2, (g) Hb3, (h) S.

### 3.5 Total Soluble Matter (TSM)

TSM is an important parameter for evaluating the water resistance and structural stability of the food-packaging materials. The TSM values of the developed biofilms were 48.114, 54.916, 60.566, and 59.431% for Hb1, Hb2, Hb3, and S, respectively, indicating that Hb1 exhibited the lowest TSM compared to the standard biofilm (S). This variation in TSM reflects differences in film stability, with Hb1 showing superior resistance to dissolution, likely because of stronger interactions between starch and the HFE. In contrast, Hb3 showed the highest TSM, suggesting weaker bonding within the matrix despite its higher density than the other films. The obtained TSM values (48–60%) are consistent with previously reported literature (38–54%), where they used Althaea rosea flowers with glycerol and bacterial nanocrystalline cellulose.^39^ Furthermore, the lower TSM observed in Hb1 suggests an improved network integrity. This behavior is consistent with enhanced hydrogen bonding and reduced polymer-chain mobility resulting from interactions between starch and hibiscus-derived phenolic compounds. Figure 5 illustrates the TSM behavior of starch-Hibiscus extract biofilms, with Hb1 showing the lowest value, followed by Hb2 and Hb3, compared with the standard film S. The reduction in TSM indicates improved intermolecular interactions within the biopolymer network, resulting in a more cohesive structure with an enhanced resistance to water.

**Figure 5:**
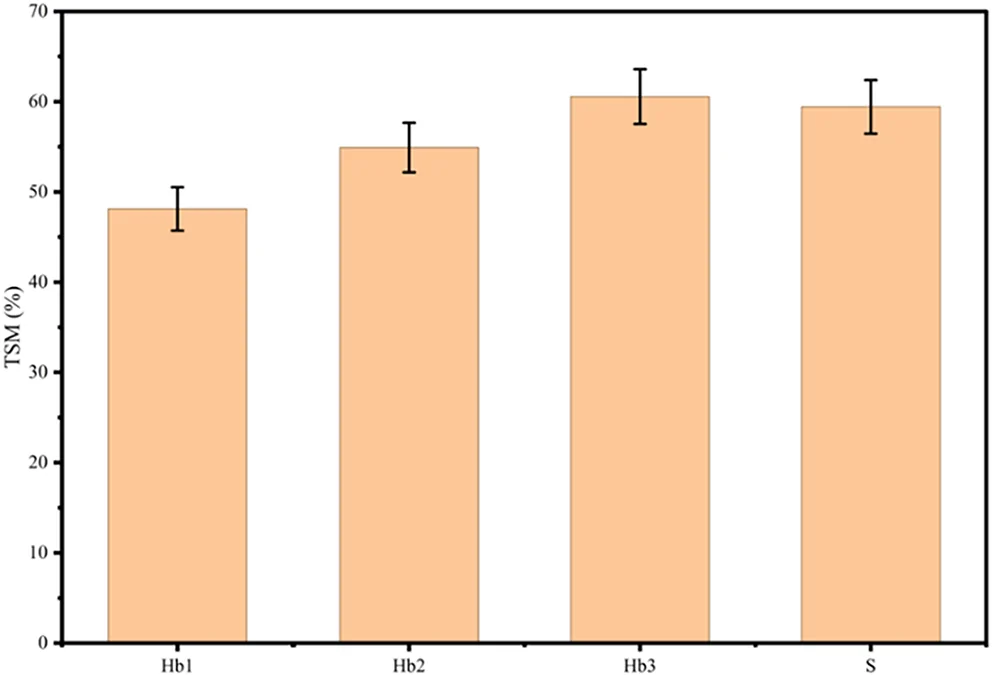
TSM of biofilms prepared using starch and Hibiscus extract.

### 3.6 Migration Test

Migration studies are essential for assessing the potential transfer of chemical constituents from packaging materials into food upon interaction with the film surface. Following immersion of the films in five different food-simulant solutions at 4 °C for 10 days, slight discolouration was observed in all samples. The λ_max_ of each simulant was determined using UV–vis spectroscopy (Table 4). The findings indicated that in simulant 1, Hb2 exhibited the highest absorbance value (2.846), suggesting greater migration. In simulants 2 and 4, Hb1 showed the highest migration, with λ_max_ values of 3.410 and 3.244, respectively. For simulants 3 and 5, the S film demonstrated the greatest migration. Overall, the UV–vis results indicate relatively lower comparative migration among the developed formulations under the investigated conditions. However, these absorbance values should not be interpreted as quantitative overall migration, as no calibration or gravimetric determination (mg/dm^2^) was performed. Among the formulations, Hb3 consistently exhibited the lowest migration across all simulants, possibly due to its dense, compact structure. In contrast, Hb1 exhibited higher migration in acidic and fatty simulants, which could be attributed to the partial release of anthocyanins and phenolic components. The S film exhibited increased migration in alcoholic and dairy simulants, likely due to weaker structural cohesion and the absence of stabilizing components. This migration analysis was performed as an initial screening approach under the conditions outlined in EU Directive 82/711/EEC to compare overall migration behavior among the different compositions. UV–vis spectroscopy was used only to confirm the presence of migrated components. The absorbance values were not converted into absolute migration quantities (mg/dm^2^) due to the complex composition of the migrated compounds and the absence of suitable calibration standards. Therefore, the results are discussed comparatively rather than quantitatively. Although migration behavior is a critical factor in evaluating food-packaging materials, limited published data are available for direct comparison. According to European guidelines, the overall migration limit for food-contact materials is 10 mg/dm^2^. Given the relatively low λ_max_ observed, particularly for Hb2 and Hb3, the developed starch-based biofilms show potential for use in secondary food-packaging. Further, it should be noted that the present migration study represents only an initial comparative evaluation of overall migration behavior. Since formaldehyde was used as a laboratory-scale crosslinking agent, quantification of residual formaldehyde and determination of its specific migration into food simulants were not performed in the present investigation. These analyses are essential for comprehensive food-safety assessment and will be included in future studies that use validated analytical techniques before any direct food-contact application is considered.

**Table 4:** Absorbance Values (Maximum) of Biofilms were Measured in Various Simulated Solutions

	λ_max_				
biofilm	simulant solution 1	simulant solution 2	simulant solution 3	simulant solution 4	simulant solution 5
Hb1	1.286	3.410	1.855	3.244	1.570
Hb2	2.846	1.562	1.818	2.923	1.232
Hb3	1.627	3.360	2.242	2.448	1.095
S	1.633	3.058	3.434	1.818	1.665

### 3.7 Scanning Electron Microscopy (SEM)

The surface morphology of the developed biofilms was investigated using scanning electron microscopy (SEM), and the micrographs are presented in Figure 6a–d. The surface morphology provides valuable insights into the compatibility between the polymer matrix and reinforcing components. Since the concentrations of HFE, citric acid, acetic acid, and formaldehyde were varied simultaneously, the observed morphological differences reflect the combined influence of extract incorporation, crosslink density, and plasticization. The following details directly influence the mechanical strength, barrier properties, and moisture resistance of the biodegradable films. The SEM image of Hb1 (Figure 6a) shows a relatively smooth, compact, and homogeneous surface with only a few microvoids and surface imperfections. The absence of significant cracks and phase separation indicates excellent compatibility among starch, refined flour, HFE, and the crosslinkers citric acid and formaldehyde.^40^ Therefore, the compact morphology suggests the formation of an interconnected polymer network through hydrogen-bonding and esterification reactions between the hydroxyl groups of starch and the carboxyl groups of citric acid, while the phenolic constituents of the HFE further strengthen intermolecular interactions.^40^ Therefore, the dense microstructure is consistent with the superior tensile strength (10.87 MPa), higher elongation at break (14.57%), lower moisture content (6.54%), and reduced total soluble matter observed for Hb1. In contrast, Hb2 (Figure 6b) showed a comparatively rougher surface with numerous platelike domains and irregular flakes distributed across the matrix. These morphological features indicate an increase in crystalline regions and a higher degree of cross-linking, attributable to increased concentrations of citric acid and formaldehyde.^41^ Although stronger crosslinking improves structural rigidity, excessive network formation restricts polymer-chain mobility, producing a stiffer material with reduced flexibility.^42^ This observation agrees well with the mechanical results, where Hb2 exhibited the highest Young’s modulus (135.69 MPa) but a significantly lower elongation at break (6.08%). Therefore, the unique surface morphology of Hb2 is consistent with its intermediate formulation, in which balanced crosslinking and extract incorporation generate a compact microstructure. Further, the SEM micrograph of Hb3 (Figure 6c) revealed a heterogeneous surface characterized by aggregated particles, irregular domains, and localized clusters dispersed throughout the matrix. These agglomerates are attributed to the combined effects of increased hibiscus extract loading and a higher crosslink density resulting from higher citric acid and formaldehyde concentrations. Therefore, the aggregation reduces the effectiveness of stress transfer across the polymer network and generates localized weak regions that adversely affect mechanical performance.^43,44^ Although Hb3 exhibited relatively good flexibility, its tensile strength remained lower than that of Hb1, indicating that excessive extract incorporation can reduce reinforcement efficiency despite increasing the availability of hydroxyl-rich bioactive compounds. However, the standard film (S) (Figure 6d) showed the most irregular surface morphology, with coarse starch granules, flaky structures, and visible discontinuities throughout the matrix. The absence of the HFE resulted in weaker intermolecular interactions and reduced cohesion within the polymer network. Further, the film exhibited the lowest tensile strength and comparatively higher moisture sensitivity and solubility. Therefore, the SEM analysis demonstrates that incorporating an optimal amount of HFE, along with other formulations, significantly improves the microstructural integrity of starch-based biofilms. Among all of the formulations, Hb1 exhibited the most uniform and compact morphology, which strongly correlates with its superior mechanical properties, lower moisture content, reduced solubility, and enhanced thermal stability. Further, excessive extract loading promoted particle aggregation and structural heterogeneity, highlighting the importance of optimizing extract concentration to achieve balanced microstructural and functional performance for biodegradable food-packaging applications.

**Figure 6:**
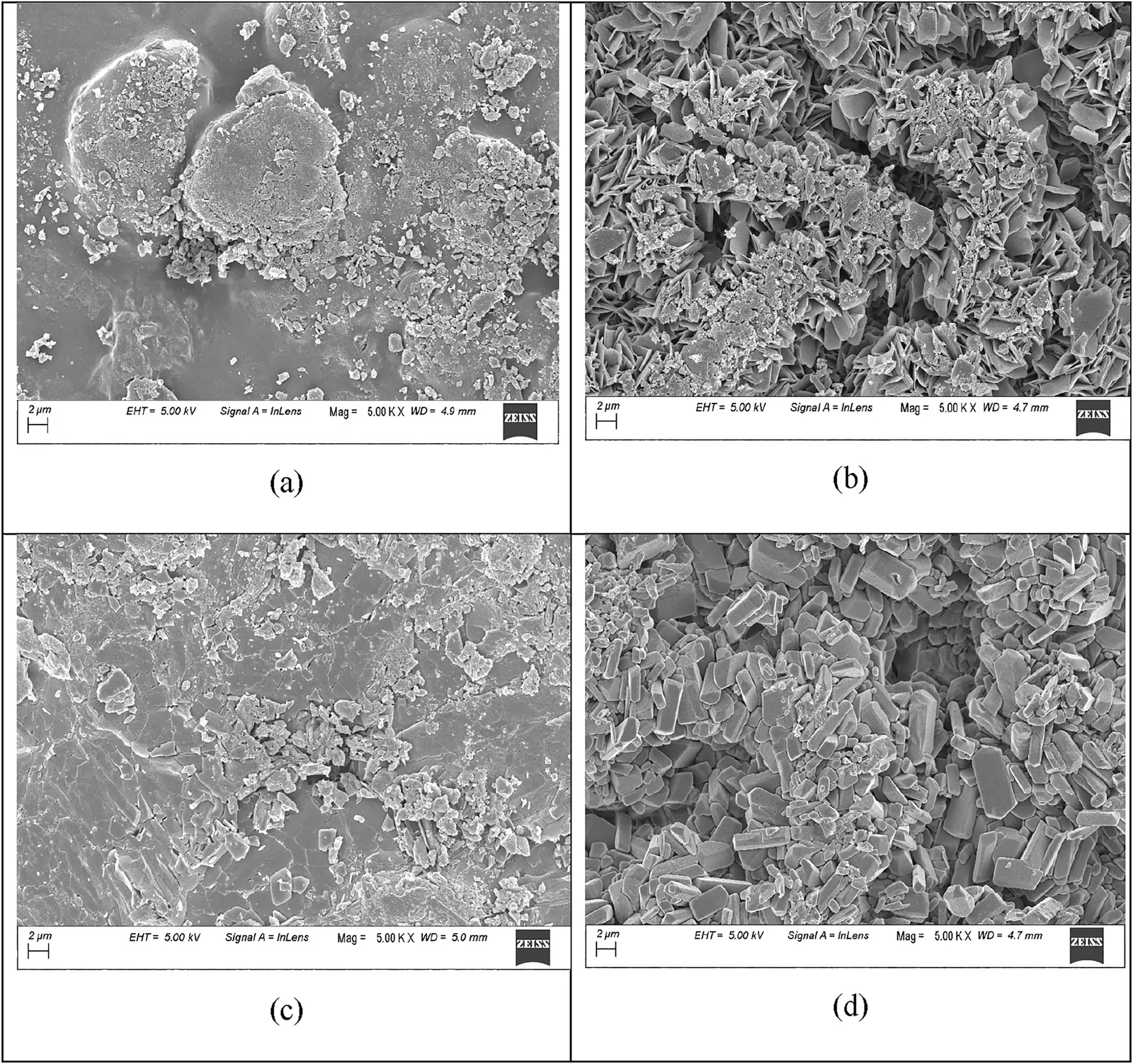
Surface morphology of biofilms (a) Hb1, (b) Hb2, (c) Hb3, (d) S.

### 3.8 FTIR Analysis

The functional characteristics of the prepared biofilms were analyzed using FTIR, and the spectra (transmittance vs. wavenumber) are presented in Figure 7a–d. The absorption band at 3353 cm^–1^ corresponds to −OH stretching vibrations associated with polysaccharides (starch) and polyphenols/flavonoids present in the hibiscus extract.^34^ The broad nature of this peak indicates strong hydrogen bonding interactions. The peaks observed in the range of 3000–2800 cm^–1^ are attributed to CH stretching vibrations of alkyl groups.^34^ The region 1680–1818 cm^–1^ represents C=O stretching vibrations of carbonyl groups, likely originating from esterified phenolic acids or organic acids such as citric or malic acid.^39^ Peaks in the 1300–1600 cm^–1^ range are associated with CH bending vibrations of alkyl groups, influenced by polysaccharide chains and ester linkages derived from plant-based compounds. Additionally, the peaks between 1000 and 1300 cm^–1^ correspond to C–O stretching vibrations of alcohol or ether groups. All films exhibited a broad OH peak at 3353 cm^–1^, with Hb1 and Hb2 showing higher-intensity, broader peaks than S, indicating increased hydroxyl group presence and stronger intermolecular hydrogen bonding due to extract incorporation.^45^ The peak at 2934 cm^–1^ corresponds to C–H stretching from aliphatic chains of starch and esterified citric acid, while the weak peak at 2584 cm^–1^ suggests the presence of carboxylic groups contributed by the extract and citric acid. The peaks at 1793 and 1702 cm^–1^ are assigned to carbonyl stretching vibrations, with Hb3 showing sharper, more intense peaks than S. This suggests enhanced esterification and crosslinking among starch, citric acid, and phenolic compounds, leading to improved structural integrity of the polymer matrix.^42,46^ The increased peak intensities at 1429 and 1326 cm^–1^ in Hb3 further confirm the contribution of aromatic polyphenols. The observed FTIR bands are consistent with functional groups commonly reported for phenolic compounds and flavonoids present in *H. rosa-sinensis* extracts. Similarly, the peaks at 1254 and 1177 cm^–1^ indicate stable C–O linkages in Hb3.^47^ Overall, Hb1 exhibited well-balanced interactions through effective hydrogen bonding and ester crosslinking, while Hb2 demonstrated strengthened ester linkages due to optimal interactions between extract and citric acid. In contrast, Hb3 showed broader, less uniform spectral features, likely due to excessive incorporation of the extract. These findings confirm that the addition of hibiscus extract significantly alters molecular interactions within the starch matrix, with HFE playing a key role in functional group modification, ester formation, and enhanced hydrogen bonding.

**Figure 7:**
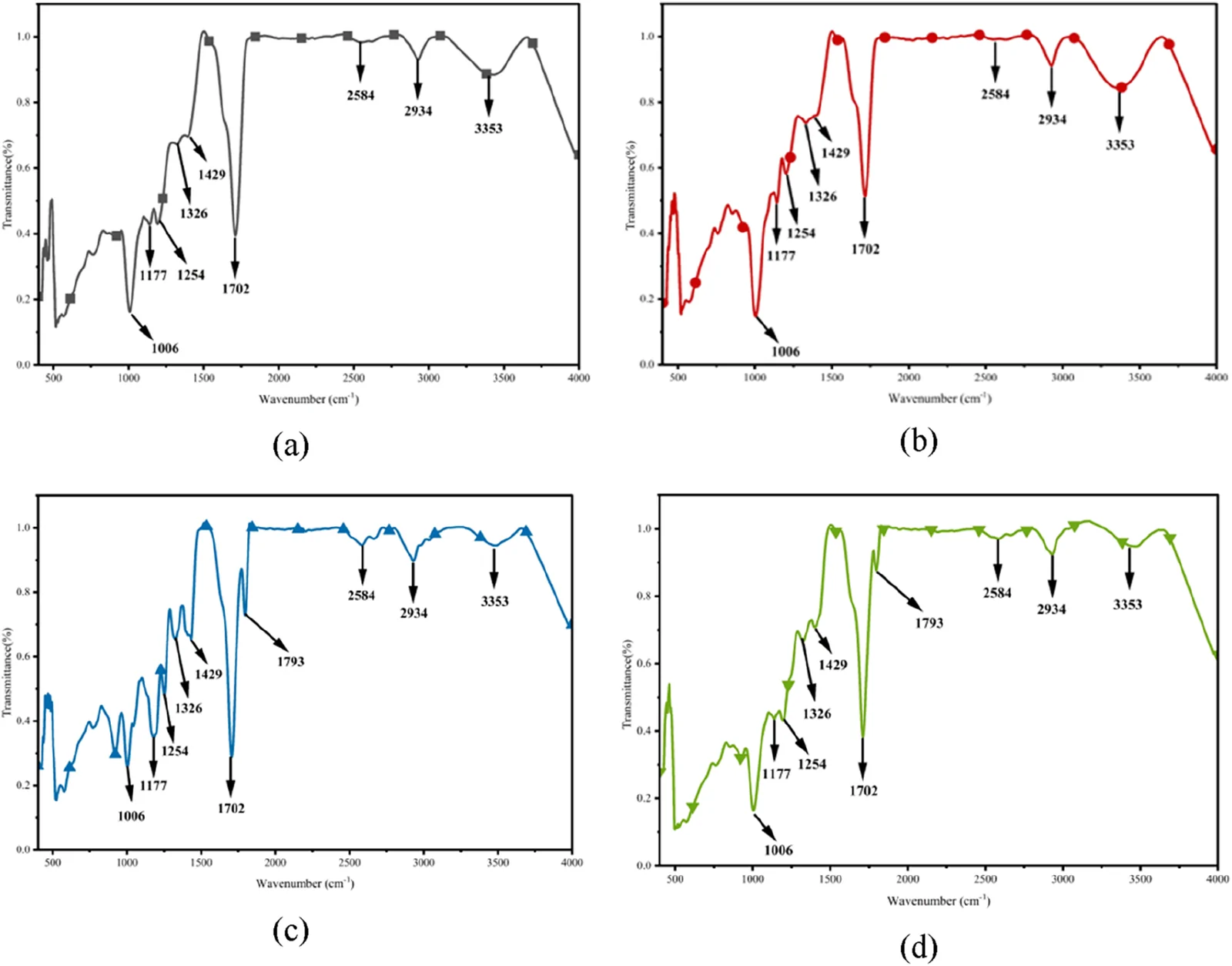
FTIR spectra of the prepared biofilm samples. (a) Hb1, (b) Hb2, (c) Hb3, (d) S.

### 3.9 Thermal Studies

Thermogravimetric analysis (TGA) was used to evaluate the thermal degradation behavior of biofilms Hb1, Hb2, Hb3, and S, as illustrated in Figure 8a–d. All samples exhibited a three-stage degradation pattern. The first stage, occurring between 80 and 200 °C, showed weight losses of 19.36, 17.21, 19.68, and 20.47% for Hb1, Hb2, Hb3, and S, respectively. This stage is mainly attributed to the evaporation of bound moisture and lighter-weight volatile substances, including water and organic residues like acetic acid.^48^ The drying phase is further supported by the broad −OH stretching band observed in the FTIR spectra, indicating the presence of abundant hydroxyl groups derived from starch and polyphenolic compounds in the HFE. The second stage of decomposition, observed between 212 and 500 °C, resulted in weight losses of 51.29, 55.34, 55.46, and 56.50% for Hb1, Hb2, Hb3, and S, respectively. This major mass loss corresponds to the primary thermal decomposition of the polymer matrix, involving the breakdown of the starch backbone and the degradation of crosslinking agents such as citric acid and phenolic or flavonoid compounds in the HFE.^49,50^ FTIR results further confirm the presence of ester, ether, and carboxyl functional groups within the biopolymer network, as evidenced by shifts or reductions in the C=O and C–O stretching bands. Notably, Hb1 exhibited a lower weight loss (51.29%) and a more gradual thermal transition, suggesting a more strongly interconnected molecular structure, as supported by FTIR analysis. In the third stage (>500 °C), biofilm degradation occurs across a wide temperature range. The mass loss in the third stage was 7.88, 14.12, 10.62, and 7.07% for Hb1, Hb2, Hb3, and S, respectively, indicating thermally stable fragments. The fragments could primarily be due to aromatic and crosslinked carbon structures incorporated by the extract, citric acid and methanal.^51^ Furthermore, the residual mass after the complete decomposition of Hb1, Hb2, Hb3, and S was 21.47, 13.33, 14.24, and 15.96%, respectively. The DTG profile of biofilms confirmed the degradation stages. In the first stage of thermal degradation (80–200 °C), a small peak arose due to the elimination of bound moisture and low-weight volatiles, such as water and acetic acid.^48^ Further, in the temperature range of 212–500 °C, the observed peak corresponds to the decomposition of the organic polymer matrix, involving the breakdown of the starch backbone and the degradation of crosslinkers such as citric acid and phenolic or flavonoid compounds present in HFE.^49,50^ Finally, in the third stage, the peak arose from aromatic and crosslinked carbon structures introduced by the HFE, citric acid, and formaldehyde.^51^ Figure 8a shows the TGA curve of Hb1 at a heating rate of 10 °C/min, with three decomposition stages: initial loss (drying) at <200 °C, followed by the main polymer degradation stage between 212 and 500 °C, yielding a mass loss of 51.29%. Figure 8b shows the TGA curve of Hb2, with three decomposition stages: the first stage (drying) at <200 °C (17.21%), followed by the main polymer degradation stage between 212 and 500 °C, with a mass loss of 55.34%. Figure 8c shows the TGA curve of Hb3 at a heating rate of 10 °C/min, showing three decomposition stages—initial loss (drying) at <200 °C (19.68%), followed by the main polymer degradation stage between 212 and 500 °C, showing mass loss of 55.46%. Figure 8d showed the TGA curve of standard (S) at 10 °C/min heating rate under an inert atmosphere, showing three decomposition stages: initial loss (drying) at <200 °C, followed by the main polymer degradation stage between 212 and 500 °C, showing mass loss of 56.50%. Therefore, the TGA and DTG analyses indicated enhanced thermal stability across all biofilms, with Hb1 showing the most significant improvement. This behavior can be attributed to hydrogen bonding (as confirmed by FTIR analysis), efficient crosslinking, and strong intermolecular interactions. The results suggest that incorporating the extract in optimal proportions produces biofilms with improved properties suitable for packaging applications. Comparable observations have also been reported in previous studies. Lee and Yeo (2021) fabricated bioplastics via gelatinization of corn starch, and TGA showed two decomposition stages. The first stage was between 34 and 272 °C, during which moisture and vinegar were evaporated, resulting in a weight loss of around 40%. In the second stage, the decomposition was between 272 and 504 °C.^48^ Andretta et al. (2019) developed starch-based films using blueberry residue. The TGA showed two stages of decomposition: the first, near 100 °C, removed moisture. The second stage occurs between 238 and 312 °C, where the main decomposition occurs, indicating that the films were highly thermally stable.^52^

**Figure 8:**
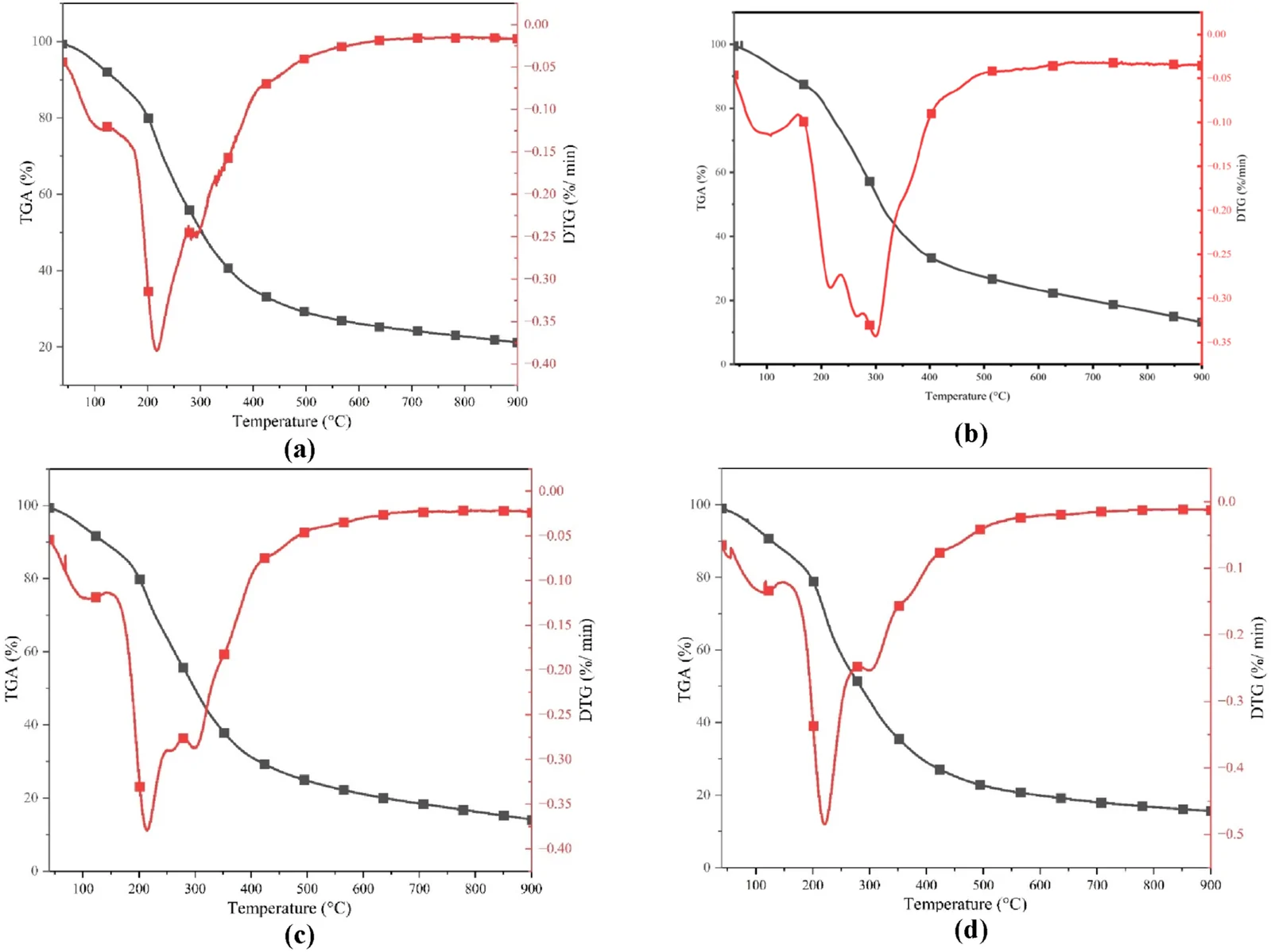
Thermal analysis of prepared biofilms. (a) Hb1, (b) Hb2, (c) Hb3, (d) S.

### 3.10 Water Contact Angle

Hydrophobicity plays an important role in food packaging as it helps limit microbial growth during storage. To assess the surface characteristics of the developed biofilms, the water contact angle (θ) formed between a droplet of distilled water and the film surface was measured. As shown in Figure 9a–d, the water contact angle (WCA) of the prepared films (Hb1, Hb2, Hb3, and S) ranged from 39 to 52°. Among them, Hb2 exhibited the highest WCA (52°), followed by Hb3 (47°) and Hb1 (39°), while the standard sample (S) showed a value (50°) close to Hb2. These results indicate that Hb1 possesses the most hydrophilic nature, whereas Hb2 demonstrates improved hydrophobicity, although it is hydrophilic in nature. The increase in WCA with increasing HFE content suggests that bioactive constituents, such as flavonoids and tannins, contribute to surface modification. This modification likely reduces water spreading by promoting additional hydrogen bonding and ester linkages within the polymer matrix. However, excessive incorporation of the HFE may reduce surface wettability. Generally, films with WCA values below 40° are more susceptible to water absorption, whereas values in the 50–60° range indicate better water resistance but may also introduce some brittleness. The obtained WCA values (39–52°) are consistent with those reported in the literature (40–60°), where they used cassava starch with glycerol.^53,54^ Based on application suitability, Hb1 may be preferred for higher adhesion and breathability, while Hb2 and S are more suitable for applications that demand improved water-barrier properties. Results confirmed that Hb2 exhibited the most balanced performance, combining moderate hydrophobicity with enhanced mechanical strength and controlled solubility.

**Figure 9:**
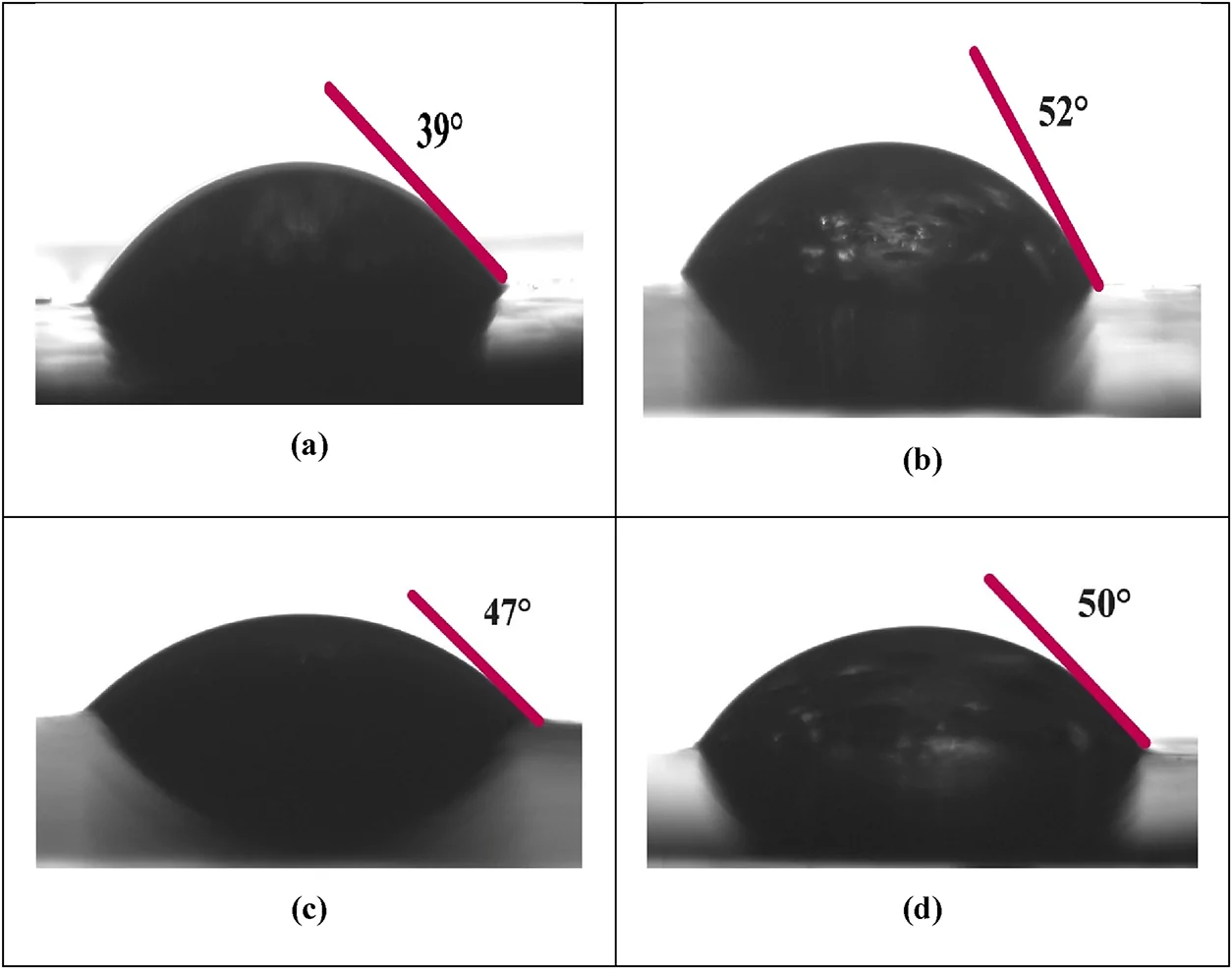
WCA of biofilms. (a) Hb1, (b) Hb2, (c) Hb3, (d) S.

### 3.11 Biodegradation Studies

All films were exposed to various environmental conditions to evaluate the biodegradation behavior of the prepared biofilms. The experiment was carried out over 30 days, with weight measurements taken at 5-day intervals to assess the biodegradability. Biofilm S consistently shows the highest degradation in soil samples across all days, peaking at around 43% on day 10. Hb1 increases notably from day 5 to day 20, reaching over 40% before slightly declining. Hb2 and Hb3 follow similar trends, with gradual increases over time and a maximum degradation of around 36–37%. Soil degradation tends to increase over time, with biofilm S showing the greatest susceptibility (Figure 10a). In all of the samples, sand degradation increases with time. Biofilm S exhibits the highest degradation, peaking above 65% at day 25 and day 30. Hb3 also shows substantial degradation, reaching over 50% by the end of the study. Hb1 and Hb2 follow similar trends, exceeding 50% degradation by day 30. Notably, all samples display significant increases between Day 15 and Day 25, indicating greater degradation in sand over time, as shown in Figure 10b. Syed Draman et al. (2025) developed bioplastics using tapioca starch (prepared and commercial) and performed degradation studies in the soil. The results showed that the prepared bioplastics degrade by 57 and 44% in 4 weeks, whereas the commercial bioplastics degrade by 12 and 11% in 6 weeks.^55^ Srinivasa Rao et al. (2022) developed bioplastics using rice starch and performed a decomposition test in soil. The results showed a volume degradability of 36-42%, revealing a similar degradation to the present study.^56^

**Figure 10:**
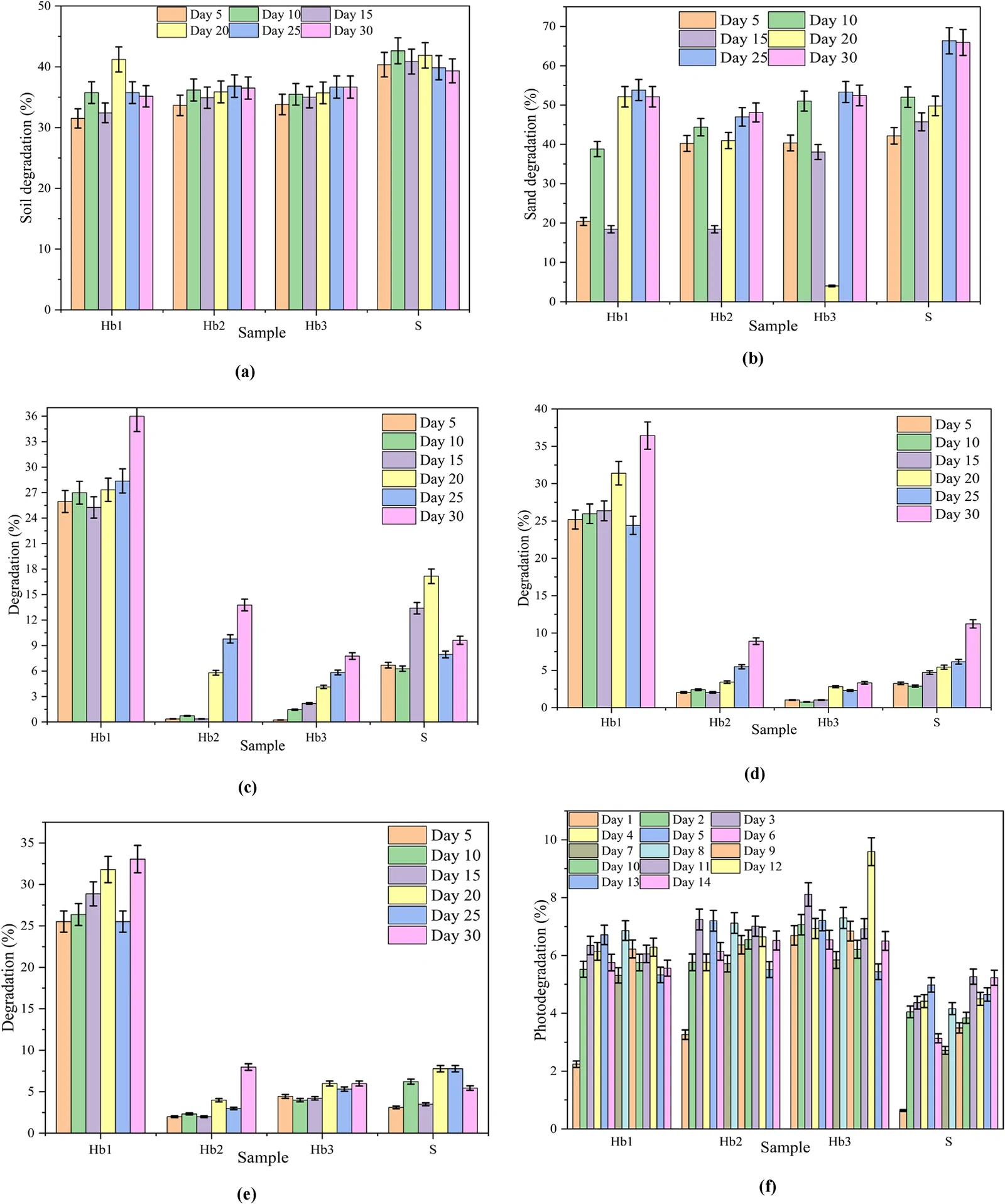
Degradation behavior of the prepared biofilms under different environmental conditions. (a) Normal soil, (b) sand, (c) distilled water, (d) tap water, (e) seawater, (f) photodegradation (under sun).

In distilled water, Hb1 shows the highest degradation, steadily increasing from about 26% on day 5 to 36% by day 30. Hb2 and Hb3 show minimal initial degradation but gradually increase, with Hb2 reaching around 13% and Hb3 reaching about 7% by day 30. Biofilm S shows a less consistent trend, peaking at approximately 17% on Day 20, then declining slightly. Hb1 degrades most rapidly and extensively, whereas Hb2 and Hb3 degrade more slowly and progressively, as shown in Figure 10c. Furthermore, the biodegradation studies of the biofilm in tap water are shown in Figure 10d. It was noticed that Hb1 exhibits the highest biodegradation, increasing steadily from around 25% on day 5 to approximately 37% by day 30 in tap water. Moreover, Hb2 and Hb3 initially show minimal biodegradation, with Hb2 reaching nearly 9% and Hb3 around 4% by day 30. Biofilm S shows a gradual increase in biodegradation from about 3 to 11% over the same period. Hb1 degrades most significantly, while the other samples exhibit slower, lower degradation rates, as shown in Figure 10d. In seawater, Hb1 shows the highest and most consistent increase in degradation, starting at around 25% on day 5 and reaching approximately 33% by day 30. Hb2 and Hb3 display moderate increases, with Hb2 peaking near 8% and Hb3 around 6%. Biofilm S shows some fluctuation, peaking at roughly 12% on Day 25 before dropping slightly on Day 30. Hb1 exhibits the most rapid degradation trend, whereas the other samples remain relatively low and variable, as shown in Figure 10e. Asmat-Campos et al. (2025) developed bioplastics using potato starch, agar, and TiO_2_ nanoparticles and performed the water degradation in seawater. The results revealed that the maximum degradation was 60% for the prepared bioplastics.^57^ Therefore, bioplastics’ degradation levels depend on the matrix’s components and their interaction with the microorganisms present in the medium as well as the conditions under which the degradation tests are carried out. Furthermore, in photodegradation, all of the samples exhibit a general upward trend. Hb3 shows the greatest increase, peaking at approximately 9% on day 12. Hb2 and Hb1 show consistent values, mostly between 6 and 7.5%, with slight fluctuations. Biofilm S shows the lowest photodegradation over the days, remaining below 6% throughout the observation period. Hb3 exhibits the most significant photodegradation, while S shows the least susceptibility, as shown in Figure 10f. Furthermore, citric acid esterification and formaldehyde-induced cross-linking increased the structural integrity of the starch matrix, the resulting material does not constitute a fully thermoset network. The degree of crosslinking is limited, and the polymer backbone remains predominantly starch-containing hydrolyzable glycosidic and ester linkages. Consequently, crosslinking primarily delays moisture diffusion and microbial access rather than prevents biodegradation, consistent with the observed weight loss during the degradation experiments.

## 4 Conclusions

Starch-based biofilms incorporating waste *H. rosa-sinensis* extract as a bioactive functional additive were successfully fabricated in the present study. The biofilm HB1 showed better mechanical strength, moisture resistance, and solubility while maintaining biodegradability and moderate transparency. FTIR and TGA data confirmed the interaction between the HFE and the starch matrix, and degradation tests in soil sand and under various water conditions validated the film’s sustainability. Thus, incorporating floral waste improved the film properties while promoting its valorization. The inclusion of HFE in starch-based biofilms offers an efficient approach for developing sustainable packaging materials, helping to mitigate issues associated with plastic pollution and organic-waste management. In addition, the starch/HFE biofilms exhibited strong potential for commercial application, owing to their cost-effectiveness and sustainability. However, the incorporation of formaldehyde, because of its toxicity, limits the use of these starch-based biofilms for direct food-contact applications. Although the developed biofilms exhibited favorable mechanical, barrier, degradation, and overall migration behavior, the inclusion of formaldehyde limits their application as direct food-contact materials. Therefore, the present formulations should be considered suitable for only secondary packaging applications. Future research will focus on replacing formaldehyde with food-grade crosslinking agents and quantifying residual formaldehyde, along with specific migration studies, using validated analytical methods to establish compliance with international food-contact regulations and ensure consumer safety. Also, standardized extract preparation followed by comprehensive chemical and biological characterization to correlate extract composition with the physicochemical and functional properties of the developed biofilm needs to be employed.

## Abbreviations

ASTM: American Society for testing and materials
CH_4_: methane
CO_2_: carbon dioxide
CR: chemical resistance
DTG: derivative thermogravimetry
EB: Elongation at break
EU: European Union
FTIR: fourier transform infrared spectroscopy
GHG: greenhouse gas
HFE: hibiscus flower extract
MC: moisture content
MT: millions of tons
RPM: revolutions per minute
SEM: scanning electron microscopy
TGA: thermogravimetric analysis
TS: tensile strength
TSM: total soluble matter
UTM: universal testing machine
UV–vis: ultraviolet visible
WVTR: water vapor transmission rate
YM: Young’s modulus
